# Harambee!: A pilot mixed methods study of integrated residential HIV testing among African-born individuals in the Seattle area

**DOI:** 10.1371/journal.pone.0216502

**Published:** 2019-05-06

**Authors:** D Allen Roberts, Roxanne Kerani, Solomon Tsegaselassie, Seifu Abera, Ashley Lynes, Emily Scott, Karen Chung, Ermias Yohannes, Guiomar Basualdo, Joanne D. Stekler, Ruanne Barnabas, Jocelyn James, Shelley Cooper-Ashford, Rena Patel

**Affiliations:** 1 Department of Epidemiology, University of Washington, Seattle, Washington, United States of America; 2 School of Medicine, University of Washington, Seattle, Washington, United States of America; 3 Department of Medicine, University of Washington, Seattle, Washington, United States of America; 4 HIV/STD Program, Public Health–Seattle and King County, Seattle, Washington, United States of America; 5 Center for MultiCultural Health, Seattle, Washington, United States of America; 6 Department of Global Health, University of Washington, Seattle, Washington, United States of America; 7 College of Arts and Sciences, University of Washington, Seattle, United States of America; Sefako Makgatho Health Sciences University, SOUTH AFRICA

## Abstract

**Background:**

African-born individuals in the U.S. are disproportionately affected by HIV yet have low HIV testing rates. We conducted a mixed methods study to assess the uptake and feasibility of a novel strategy for integrating HIV testing into residential health fairs among African-born individuals in Seattle, WA.

**Methods:**

From April to May 2018, we held six health fairs at three apartment complexes with high numbers of African-born residents. Fairs included free point-of-care screening for glucose, cholesterol, body mass index, blood pressure, and HIV, as well as social services and health education. The health fairs were hosted in apartment complex common areas with HIV testing conducted in private rooms. Health fair participants completed a series of questionnaires to evaluate demographics, access to health services, and HIV testing history. We conducted 18 key informant interviews (KIIs) with health fair participants and community leaders to identify barriers to HIV testing among African-born individuals.

**Results:**

Of the 111 adults who accessed at least one service at a health fair, 92 completed questionnaires. Fifty-five (61%) were female, 48 (52%) were born in Africa, and 55 (63%) had health insurance. Half of African-born participants accepted HIV testing; all tested negative. The most common reasons for declining testing were lack of perceived risk for HIV and knowledge of HIV status. We identified a high prevalence of non-communicable diseases (NCDs) among health fair participants; among those tested, 77% (55/71) were overweight/obese, 39% (31/79) had blood pressure > 140/90 mmHg, and 30% (22/73) had total cholesterol > 200 mg/dL. KIIs identified community stigma and misinformation as major barriers to HIV testing among African-born individuals.

**Conclusions:**

Residential health fairs are a feasible method to increase HIV testing among African-born individuals in Seattle. The high prevalence of NCDs highlights the importance of integrating general preventive services within HIV testing programs in this population.

## Introduction

Foreign-born individuals comprise an estimated 16% of people newly diagnosed with HIV in the U.S. [1] In King County, Washington, African-born individuals account for 2% of the population yet 10% of new HIV diagnoses [2,3]. Migrant populations face individual, social, and structural barriers to HIV testing in the U.S, including stigma, language barriers, limited financial resources, and insufficient provider cultural competency [4]. As a result, African-born individuals with HIV commonly present late to care [5]. In King County in 2016, 34% of foreign-born blacks received an AIDS diagnosis within one year of their HIV diagnosis [6]. However, once linked to care, retention and treatment outcomes among foreign-born individuals are similar or better than those of US-born persons [7–9].

Early HIV diagnosis and treatment prevents morbidity, mortality and onward transmission [10–12]. Therefore, identifying HIV testing models that effectively engage African-born individuals is important for public health programs. Community-based HIV testing provides services outside of health facilities and is sometimes integrated with non-communicable disease screening to increase coverage and decrease stigma. In sub-Saharan Africa, community-based testing has been shown to achieve high coverage, identify HIV-positive people at higher CD4 counts, and reach populations who are less likely to seek care at health facilities [13]. Community-based HIV testing programs for African-born populations in the U.S. have been primarily located in the northeast, with varying levels of uptake [14–16]. Local studies are needed to test if community-based HIV testing can decrease the barriers to testing and increase uptake.

To address this gap, our team designed and piloted Harambee!, a residential HIV testing intervention consisting of preventive health fairs located at Seattle area apartment complexes with high numbers of African-born residents. We partnered with Center for MultiCultural Health, a community-based organization that provides HIV prevention services to the local African-born community, and with volunteer medical service providers from the University of Washington Schools of Medicine, Pharmacy, and Dentistry. To evaluate the feasibility and acceptability of Harambee!, we conducted a mixed-methods study. The specific objectives of this study were to: a) assess the uptake, test results, and cost of services provided in this model, and to b) qualitatively explore barriers to and facilitators of HIV testing among African-born individuals.

## Methods

We conducted a prospective, mixed methods study to assess the uptake and feasibility of integrating HIV testing into residential health fairs among African-born individuals in Seattle, WA.

### Selection and advertising of health fair locations

In April and May 2018, we conducted a total of six health fairs on two consecutive Saturdays at three apartment complexes. The apartment complexes were identified through our community partner’s social network and selected to represent geographically diverse neighborhoods within the greater Seattle area. With the support of apartment complex managers, we conducted site visits 2–3 months before the planned health fair to assess the availability of communal spaces for service provision and private rooms for HIV testing. Study staff advertised health fairs before the planned event by posting flyers in five languages on unit doors, in communal spaces, or in mailboxes. In addition, apartment complex managers sent mass text messages alerting residents of the fairs when possible. Study staff posted signage on the day of the event at common entrances and handed out flyers in-person while canvassing. Each health fair lasted between four and six hours.

### Service provision

We recruited volunteer service providers to offer point-of-care screening and counseling for blood pressure, body mass index (BMI), non-fasting blood glucose, total cholesterol, high density lipoprotein (HDL), and HIV at each health fair. Glucose, cholesterol, and HDL results were obtained using the CardioChek™ Professional Analyzer (PTS Diagnostics, Indianapolis, USA) while HIV tests were obtained with the INSTI HIV-1/HIV-2 Antibody Test (bioLytical Laboratories, Richmond, Canada). All services were provided free of charge and anonymously. Participants received a record of their test results, and participants with abnormal results and no existing primary care provider were offered assistance in setting up an appointment at a local primary care clinic. Several health fairs also included dental exams, mental health information, social services, and children’s activities. Professional medical interpreters were available telephonically for all languages, and each health fair included volunteers fluent in Spanish, Amharic, and Tigrinya to assist in fair navigation or in-person interpretation if requested. A licensed physician provided supervision at each health fair. All the health fair services were available to everyone attending the health fair regardless of their participation in the study, country of birth, or residence status at the apartment complex.

### Informed consent

All individuals 18 years of age or older attending the health fair were approached to participate in two study questionnaires. Participants provided verbal consent, and a health fair identification number was assigned to each participant to maintain anonymity. The informed consent documents were written in English and then translated into Spanish, Somali, Amharic, and Tigrinya. All translations were done by a professional translation service and were reviewed for content and accuracy by native speakers. Potential participants who did not read one of the five languages in which we had prepared materials had the option to ask another individual of their choosing to provide in-person interpretation. Potential participants were briefly oriented to the study goals and asked to read the consent documents. Questions or concerns were addressed by study staff.

### Data collection

All study participants were invited to complete two questionnaires. The first questionnaire was offered at health fair entry and consisted of demographic and general health care questions, including the Patient Health Questionnaire-2 (PHQ-2), that each participant self-completed [17]. The second questionnaire was administered in a private room by trained research staff and addressed HIV testing history and behavioral risk factors. All participants who completed the second questionnaire and who were not known to have HIV were offered HIV testing. We used a health fair identification number to link questionnaire data to screening test uptake and results. Participants who completed the questionnaires, regardless of which services they accessed, received a $10 reimbursement. Study data were collected on paper forms and later entered and managed using REDCap [18].

We also conducted a series of key informant interviews (KIIs) with health fair participants and community leaders to identify barriers to HIV testing and general preventive health care among African-born individuals. We obtained a purposeful sample at each health fair by identifying 2–3 candidates to interview who represented a diversity of birth place, gender, and occupation and were conversant in the interviewer’s language (primarily English, but occasionally Spanish or Amharic). All health fair participants approached for interviews agreed to participate. The interview candidates were given a separate informed consent document and provided verbal consent. We used snowball sampling to identify community leaders for KIIs. First, our community partners identified initial informants in the health sector among the African-born communities in Seattle. For in-person interviews, participants were read the informed consent script and provided verbal consent. For phone interviews, the informed consent and interview guide documents were sent via email to the interview candidate to review ahead of the scheduled interview. All approached participants agreed to interview with us, with one person opting to provide her responses to the interview guide via email. Once we interviewed an individual, we asked that person to recommend another 1–2 people we should consider interviewing and their contact information. We attempted to match the interviewer with a native-speaking interviewee when possible. Four study staff conducted all the interviews, with the large majority being conducted by one person (SA). The interviews lasted generally 30–40 minutes and no reimbursements were provided. We took detailed hand notes during the interview, attempting to capture key quotes verbatim, and transcribed the notes into an electronic document. We stopped conducting KIIs once saturation of themes was reached.

### Data analysis

We estimated total health fair attendance by dividing the number of people accessing any service by the estimated number of adults residing in the apartment complex. The denominator was obtained directly from apartment complex managers for two sites and was estimated at the third assuming an average household size of 2.1 [19]. Among those enrolled in the research study, we calculated descriptive statistics for participant demographics, access to health care indicators, HIV testing history, and attitudes toward people living with HIV. As HIV testing was offered concurrently with the second questionnaire (concerning HIV testing history and risk behaviors), we calculated HIV testing uptake as the percentage of study participants completing the second questionnaire who accepted HIV testing. We compared test uptake across sociodemographic characteristics and HIV testing history and used chi-square tests to assess statistical significance. We classified BMI, blood pressure, and blood testing results based on national guidelines [20–23]. We report results for general health care, HIV, and non-communicable disease (NCD) outcomes stratified by place of birth (US-born, African-born, or other foreign-born).

We conducted an ingredients-based micro-costing study to estimate the incremental economic cost of adding HIV testing to residential health fairs. Costs for HIV testing included test kits, test supplies, transportation, personnel time, and interpreter costs. As HIV testing was conducted by volunteers, we estimated personnel costs using wages paid to outreach HIV testers employed by the county health department. We assumed a cost of $2/minute for donated telephonic interpreter services [24]. Costing was conducted from the provider perspective and reported in 2018 United States dollars (USD). We did not estimate the overall cost of the health fairs given multiple volunteer services.

For the KIIs, we used thematic analysis to code the field note transcripts. One study team member coded all the field note transcripts and another study team member reviewed the coding. When discrepancies arose, we used discussions to arrive at consensus. Once coding of the initial ten transcripts was completed, we arranged the codes into larger, thematic domains followed by both convergent and divergent subthemes and illustrative quotations. This coding strategy was iteratively improved upon until final coding of all field note transcripts was complete.

### Ethics statement

Ethical approval for study and oral informed consent procedures was obtained from the University of Washington Human Subjects Division. As study participation was anonymous, oral consent, responses to study questionnaires, and test results were documented using a unique identification numbering system.

## Results

### Health fair attendance

One hundred eleven people accessed at least one service across the six health fairs, with a median of 18 people attending each fair. Combined attendance across the two fairs from each site was 16% (26/160), 11% (53/489), and 4% (32/796). Fifty-two people tested for HIV. Blood testing for glucose and cholesterol were the services most frequently accessed (n = 103), followed by blood pressure (n = 95) and BMI (n = 90).

### Characteristics of study participants

Ninety-two people completed study questionnaires. The majority of attendees were aged 30–49 (52%), women (61%), and identified as Black, African-American or African (59%, **Table 1**). Amharic (49%) and English (40%) were the most common languages spoken at home, and 41% of participants were born in Ethiopia. Twenty-three percent had immigrated to the US in the past five years. African-born and other foreign-born participants were less likely to have health insurance or a primary health care provider compared to US-born participants (**Table 2**). Most health fair participants (68%) reported having undergone a routine checkup in the last year. African-born (69%) and other foreign-born (38%) participants were less likely to have ever tested for HIV compared to US-born participants (90%).

**Table 1:** Demographics of health fair participants completing study questionnaires, King County, WA, 2018 (N = 92).

Variable	n	%^†^
**Age**		
< 30	7	9
30–39	23	30
40–49	17	22
50–59	18	24
> 59	11	15
**Gender**		
Men	34	38
Women	55	61
Other	1	1
**Race**		
Black/African-American/African	53	59
Hispanic/Latinx	19	21
White	6	7
Asian or Asian American	5	6
Pacific Islander or Native Hawaiian	2	2
Multiple	1	1
Other	4	4
**Religion**		
Christian or Catholic	68	76
Muslim	13	15
No affiliation/Other	8	9
**Currently employed**		
Yes	47	53
No	42	47
**Education**		
None	4	5
Primary	14	16
High/secondary	36	42
Some college, but no degree	17	20
College degree	13	15
Other	2	2
**Language spoken at home**^‡^		
English	44	49
Amharic	36	40
Spanish	16	18
Tigrinya	9	10
Somali	4	4
Other	17	18
**Country of birth**		
Ethiopia	38	41
US	20	22
Mexico	15	16
Other (Africa^§^)	10	11
Other (outside of Africa ^‖^)	9	10
**Immigrated within last five years**		
Yes	15	23
No	51	77
**Traveled outside of US within last five years**		
Yes	26	29
No	63	71

^†^Missing values excluded from percentages. Number missing for each variable was: Age (16), Gender (2), Race (2), Religion (3), Currently employed (3), Education (6), Language spoken at home (2), Country of birth (1), Immigrated within last five years (6), Traveled outside of US within last five years (3)

^‡^Multiple languages could be selected

^§^Other African countries included Somalia (3), Eritrea (2), Sierra Leone (2), Sudan (1), Kenya (1)

^‖^Other non-African countries included Bangladesh (3), Afghanistan (2), Poland (2), El Salvador (1), and Belize (1)

**Table 2:** Prior engagement with medical care among health fair participants completing study questionnaires, stratified by birthplace, King County, WA, 2018 (N = 92).

Variable	US-Born n (%)	African-Born n (%)	Other Foreign-Born n (%)	Total n (%)
**Currently has health insurance**				
Yes	16 (80)	30 (63)	9 (38)	55 (60)
No	3 (15)	16 (33)	12 (50)	31 (34)
Unsure/Missing	1 (5)	2 (4)	3 (13)	6 (7)
**Has primary care provider**				
Yes	11 (55)	21 (44)	13 (54)	45 (49)
No	7 (35)	17 (35)	8 (33)	32 (34)
Unsure/Missing	2 (10)	10 (21)	3 (13)	15 (16)
**Last routine health checkup**				
Less than one year ago	15 (75)	35 (73)	13 (54)	63 (68)
1–2 years ago	3 (15)	5 (10)	4 (17)	12 (13)
2–5 years ago	0	1 (2)	4 (17)	5 (5)
More than 5 years ago	0	0	1 (4)	1 (1)
Never	0	6 (13)	0	6 (7)
Unsure	2 (10)	1 (2)	2 (8)	5 (5)
**Ever tested for HIV**				
Yes	18 (90)	33 (69)	9 (38)	65 (71)
No	2 (10)	11 (23)	14 (58)	22 (24)
Unsure/Missing	0	4 (8)	1 (4)	5 (5)
**Ever lived in the same house as someone with TB**				
Yes	1 (5)	2 (4)	0	3 (3)
No	16 (80)	32 (67)	22 (92)	70 (76)
Unsure/Missing	3 (15)	14 (29)	2 (8)	19 (21)
**Ever taken medications for TB treatment**				
Yes	1 (5)	3 (6)	0	4 (4)
No	18 (90)	31 (65)	22 (92)	71 (77)
Unsure/Missing	1 (5)	14 (29)	2 (8)	17 (18)

TB = tuberculosis

### Uptake of HIV testing

Among 87 study participants who completed the HIV testing and risk behavior questionnaire, 49 (56%) accepted point-of-care HIV testing. Half (22/44) of African-born participants chose to test; of those who accepted testing, 22% had never tested before, 43% had not tested in the last 5 years, and 64% had never tested in the USA before (S1 Table). Uptake was higher among women (63%) compared to men (44%) and among people who had not tested in the last year (59%) compared to those who had (38%). None of the predictors that we evaluated (age, gender, birthplace, HIV testing history, number of sexual partners in the last 12 months, having a friend or family member known to have HIV, or having health insurance) were statistically significantly associated with accepting HIV testing. The most common reasons provided for declining HIV testing among those who responded included lack of perceived risk (16/33, 48%) and knowledge of HIV status (14/33, 43%).

### Screening results

Overall, 77% of participants were overweight or obese (**Table 3**). Seventy-two percent of participants had elevated blood pressure above 130/80 mm Hg and 39% had blood pressure above 140/90 mm Hg. Fewer African-born participants had blood pressure above 140/90 than other participants (30% vs. 48%, respectively), but more African-born participants had total cholesterol above 200 mg/dL than other participants (38% vs. 21%, respectively). Ten percent of participants screened had non-fasting glucose levels greater than 200 mg/dL. Seven of 80 participants (9%) who completed PHQ-2 questionnaires had positive screens for depression. All participants screened for HIV tested negative.

**Table 3:** Health screening results among health fair participants completing study questionnaires, stratified by birthplace, King County, WA, 2018.

Variable	US-Born N (%)	African-Born N (%)	Other Foreign-Born N (%)	Total N (%)
**BMI (kg/m**^**2**^**)**				
Underweight (< 18.5)	0	0	0	0
Healthy (18.5–24.9)	1 (6)	11 (31)	4 (21)	16 (23)
Overweight (25–29.9)	3 (19)	15 (42)	6 (32)	24 (34)
Obese (> 30)	12 (75)	10 (28)	9 (47)	31 (44)
**Blood Pressure (mm Hg)**				
< 130/< 80	2 (13)	13 (33)	7 (30)	22 (28)
130-139/80-89^†^	5 (31)	15 (38)	6 (26)	26 (33)
>140/>90	9 (56)	12 (30)	10 (44)	31 (39)
**Non-fasting Glucose (mg/dL)**				
Normal (< 200)	11 (85)	38 (95)	17 (85)	66 (90)
Elevated (200+)	2 (15)	2 (5)	3 (15)	7 (10)
**Total Cholesterol (mg/dL)**				
Normal (< 200)	12 (92)	25 (63)	14 (70)	51 (70)
Elevated (200+)	1 (8)	15 (38)	6 (30)	22 (30)
**HDL (mg/dL)**				
Normal (40+)	9 (69)	31 (80)	16 (80)	56 (78)
Low (< 40)	4 (31)	8 (20)	4 (20)	16 (22)
**PHQ-2 (score)**				
Negative (< 3)	17 (94)	45 (96)	18 (82)	80 (92)
Positive (3+)	1 (6)	2 (4)	4 (18)	7 (8)
**HIV**				
Negative	10 (100)	22 (100)	16 (100)	48 (100)
Positive	0	0	0	0

^†^Includes individuals with either systolic between 130 and 139 or diastolic between 80–89, or both.

### Costing

Assuming an hourly wage of $28 (obtained from a local outreach HIV testing program), the average personnel cost for two HIV testers at each health fair was $392. The supplies cost for each HIV test was $9.78, with the HIV test ($8) comprising the majority. In total, the total incremental cost of HIV testing across the six health fairs was $3,254, corresponding to $63 per HIV test conducted. The largest cost components included personnel (72%), supplies (16%), and interpretation (9%).

### Qualitative results

We conducted KIIs with 18 people, ten of whom were women and 14 of whom were African-born. When asked about barriers among African-born individuals to testing for HIV in the Seattle area, the most common theme identified by KII participants was stigma (**Table 4**). Fear of social isolation and shaming from other community members was frequently mentioned. Others noted how the perception of HIV diagnosis as a death sentence can worsen stigma and increase fear of getting tested. Some participants also mentioned perceived stigma from health care providers. Other barriers identified included misinformation about HIV risk and HIV testing services as well as prioritizing work over health.

**Table 4:** Barriers to and facilitators of HIV testing: qualitative findings.

Domain: Barriers to getting tested for HIV		
Major theme	Subtheme	Indirect Quote
Stigma	Community stigma and social isolation	We are socially integrated, so people are afraid the community will know about our status, . . .[and] many people in my community care a lot about what people know and think about them. [If someone is diagnosed with HIV], their social engagement will be affected, and many families will not want their children to socialize with that particular person. In general stigma is an issue. *54-year old Ethiopian male taxi driver*
		[Those sharing their HIV positive diagnosis] will be shamed, isolated, victim of gossip . . .not official denial of participation but people will isolate them. *43-year old Ethiopian female educator*
		A member of my community in their right mind will not publicly share a positive HIV diagnosis. They will be isolated, people will not engage with him/her. People in the community will be uneasy around them. They wouldn’t be invited to eat with others from the same plate. *24-year old Kenyan female student*
	Associate HIV diagnosis with death sentence	Another unique aspect is the knowledge the community has about the disease—it is still scary and considered as death sentence—so that worsens stigma . . .So [if someone in my community finds out about someone’s positive HIV status] they will be shocked, as the impression the community has about the disease and the reality about the disease are different. *58-year old Eritrean male health professional*
	Health systems	I think the unique aspect that can worsen stigma is the difficulty of creating rapport with health providers. But once rapport and trust is created with the health care providers, stigma will be less of an issue . . .[But the] absence of trust between community members and health care providers is a major issue. *58-year old Eritrean male health professional*
Lack of awareness	Knowledge about HIV, risk perception	Misconception about the disease makes people to be scared about their disease and not considering yourself at risk . . . *37-year old Ethiopian male health professional*
		People think it won’t happen to them, they consider themselves exceptional *26-year old Ethiopian female student*
		There are also gender differences in expectations…worse for women to be HIV+ because we are already expected to not have many partners, and people think that they have had many partners if HIV+, even if they have only had one partner and got it from them. *18-year old Ethiopian female student*
	HIV-related services	Most people don’t know where to go, especially to places that offer free HIV testing. Most people think it costs a lot of money to get HIV testing. *38-year old female Ethiopian community health worker*
Prioritizing work over health		Being busy at work and lack of access to services like not having insurance. *41-year old Ethiopian male airport attendant*
**Domain: Facilitators for improving HIV testing rates**		
Increasing education or awareness		Many are scared to know their result because they are so intimidated by the disease…Education and creating awareness is the way to solve it. *32-year old Somali female health professional*
Location of testing		Maybe it’s too much, but maybe go to home of person . . . *56-year old Mexican female apartment manager*
		If the testing is provided in a community setting, there will be some issues about their neighbors knowing specially if they are from the same community, but I believe the benefits outweigh negatives as long as the tests are done in private rooms. *54-year old Ethiopian male taxi driver*
Health systems	Culturally appropriate services	Culturally appropriate services, developing support groups which has social workers, lawyers, volunteer, donors and community members . . . Friendly, short, high confidentiality, interventional, with proper pre and post-test counseling. *58-year old Eritrean male health professional*
	Integrating HIV testing into routine care to destigmatize it	Other healthcare services will be welcome but stigma can potentially be an issue with HIV so integrating them may help. *60-year old Ethiopian female health professional*
		It would help if they make HIV testing more as a regular check-up. Don’t put a “sticker” on it [motioning to shirt where a nametag would be], like this is special. Should be regular like cholesterol–you test cholesterol, you test HIV. We as society make stigma by not treating HIV testing as part of routine care. *56-year old Mexican female apartment manager*

When asked about factors that facilitate HIV testing among African-born individuals, KII participants commonly identified making HIV testing more accessible by providing testing in community locations or in homes. Several participants identified confidentiality and privacy as key features of community-based testing. Providing culturally competent care and integrating HIV testing into routine services were also mentioned. Other facilitators included increasing education and awareness about treatment availability and effectiveness.

## Discussion

In this study, we demonstrated the feasibility of integrating HIV testing into residential health fairs designed for African born individuals. HIV testing in this setting was acceptable, with over half of all participants, and 50% of African-born participants, electing to test. We identified stigma and misinformation as critical barriers to HIV testing in African-born communities. Additionally, we found high prevalence of obesity, high blood pressure, hyperglycemia, and hyperlipidemia among all health fair attendees, regardless of birthplace.

Few reports of HIV testing programs designed for African-born individuals in high income countries have been published previously. We achieved relatively high uptake with over 50% of study participants undergoing HIV testing. In comparison, an oral fluid HIV testing program at community venues among African-born individuals in Belgium achieved 18% uptake out of 780 offered testing, while an HIV education and testing intervention through a soccer tournament in Massachusetts was only able to test 2% of about 500 individuals attending [25,26]. However, a large community clinic program in Philadelphia with substantial health department support tested 92% of 4152 health fair participants over four years [15]. The authors identified provider cultural competence, early community leader engagement, bundling HIV testing with other services, and scheduling flexibility as critical ingredients to achieving high uptake. These features are important considerations for health programs implementing community testing models.

We found high levels of obesity (44% BMI > 30), blood pressure (39% > 140/> 90 mmHg), non-fasting hyperglycemia (10% > 200 mg/dL), and hypercholesterolemia (30% > 200 mg/dL) among health fair attendees. In addition, participants had low levels of insurance coverage (60%), and only half reported having a primary care provider. Our results are consistent with national-level estimates of obesity (29%), hypertension (28%), diabetes (9%), and health insurance coverage (70%) among foreign-born blacks [27,28]. Integrating NCD screening may be especially important due to the growing burden of NCDs globally and in sub-Saharan Africa specifically [29–31]. Our results caution that isolated HIV testing programs in this population may represent missed opportunities for cardiometabolic screening and linkage to primary care. In addition, our qualitative results suggest that integrating HIV into these less stigmatizing medical conditions may have promoted the high uptake of HIV testing in our study.

We estimated that an average of 10% of the adults living in the apartment complexes attended our fairs. Attendance varied by apartment complex, possibly owing to differences in demographics, community engagement by apartment complex managers, visibility of the public space, or timing. While community-based strategies have previously been shown to increase engagement in care for men, attendance and acceptance of HIV testing at our health fair were higher among women than men (though the difference was not statistically significant) [13]. Further efforts are needed to effectively engage men into HIV testing and preventive health care. While our reliance on volunteer staff necessitated weekend health fairs, future efforts should explore alternative timings, such as weekday evenings, that may better accommodate schedules for men. Embedding health fairs into larger promotional events, such as concerts or festivals, may be another strategy to increase attendance. Strong partnerships with community involvement in the design, promotion, and implementation is critical to the success of these events.

At $63, the incremental cost of HIV testing was similar to other community-based programs for mobile testing in key populations [32]. These results support the feasibility of integration of HIV testing into community-based health fairs. Personnel comprised the largest fraction of overall cost (72%). The training of our service providers necessitated using separate personnel for provision of NCD screening services and HIV testing. Programs may find that using the same health worker to offer all services could further increase uptake of HIV testing and benefit from economies of scope.

Our qualitative results highlight stigma and misinformation as critical barriers that limit HIV test uptake among African-born individuals. HIV-related stigma is common among African-born communities in high income countries and has been previously identified as a factor that hinders HIV testing and disclosure in this community [33–37]. While only two people mentioned confidentiality as a reason for declining testing during the health fairs, it is possible that people who fear HIV testing were less likely to attend the health fairs or participate in the study. Future efforts to pilot HIV education and stigma reduction interventions are needed to address these barriers.

Our study has several limitations. Our measure of HIV test acceptance may be an overestimate if non-study participants were less likely to choose to be tested for HIV compared to study participants. As a pilot project, the results of this program may not be generalizable due to the small sample size. No participants screened positive for HIV, which could be due to small numbers or that health fair participants had low risk of HIV infection. Other testing models, including social network strategies, incentivized testing, and distribution of HIV self-testing kits, should be piloted in this community. We did not ascertain detailed HIV risk factor information from study participants, making it difficult to assess the underlying HIV risk of participants. However, specific behavioral risk factors that predict HIV acquisition are not well understood for African-born immigrants, who come from a generalized epidemic setting and often report low numbers of partners [1,3,38]. Last, African-born communities in high-income countries exhibit substantial heterogeneity in country of origin, access to health services, and culture. Close partnership with local community-based organizations is essential to tailor interventions towards community needs. Despite these limitations, the results of this pilot program suggest a path forward for efforts seeking to increase HIV testing among African-born individuals.

## Conclusions

In conclusion, we successfully implemented a novel community-based, integrated HIV testing intervention that was acceptable among African-born individuals. The high prevalence of NCDs among health fair participants highlights the importance of integrating general preventive health services into HIV testing programs in this community. Given that health department funding is typically siloed across health conditions, with HIV funding restricted primarily to HIV prevention and care, coordination across organizations and agencies will be necessary to effectively address the preventive health needs of African-born individuals.

## Supporting information

S1 Table Uptake of HIV testing among health fair participants completing HIV testing and risk behavior questionnaire, King County, WA, 2018 (N = 87). (DOCX)

S1 File Study Questionnaires and Interview Guides. (PDF)
