# Supplementary material for: Harambee!: A pilot mixed methods study of integrated residential HIV testing among African-born individuals in the Seattle area
Source: PLoS One. 2019 May 6;14(5):e0216502. doi: 10.1371/journal.pone.0216502 (PMC6502314; doi:10.1371/journal.pone.0216502)
Supplement: S1 Table — (DOCX) [file pone.0216502.s001.docx]

**S1 Table: Uptake of HIV testing among health fair participants completing HIV testing and risk behavior questionnaire, King County, WA, 2018 (N = 87)**

| **Variable** | **Accepted**  **HIV Test** | **Declined**  **HIV Test** | **% Accepted** | ***p*-value^†^** |
| --- | --- | --- | --- | --- |
| **Age** |  |  |  |  |
| < 35 | 7 | 11 | 39 | 0.32 |
| 35-49 | 16 | 10 | 62 |  |
| 50+ | 15 | 12 | 56 |  |
| **Gender**^‡^ |  |  |  |  |
| Men | 14 | 18 | 44 | 0.12 |
| Women | 33 | 19 | 63 |  |
| **Birthplace** |  |  |  |  |
| US | 10 | 10 | 50 | 0.27 |
| Africa | 22 | 22 | 50 |  |
| Other | 16 | 7 | 70 |  |
| **Previously tested for HIV** |  |  |  |  |
| Yes | 12 | 10 | 55 | 1 |
| No | 36 | 29 | 55 |  |
| **Tested for HIV in last year** |  |  |  |  |
| Yes | 6 | 10 | 38 | 0.22 |
| No | 34 | 24 | 59 |  |
| Unknown^§^ | 8 | 5 | 62 |  |
| **Number of sexual partners in last year** |  |  |  |  |
| 0 | 14 | 11 | 56 | 0.34 |
| 1 | 30 | 25 | 55 |  |
| 2+ | 3 | 0 | 100 |  |
| **Friend or family member known to be HIV+** |  |  |  |  |
| Yes | 7 | 3 | 70 | 0.49 |
| No | 41 | 35 | 54 |  |
| **Health insurance** |  |  |  |  |
| Yes | 26 | 26 | 50 | 0.23 |
| No | 19 | 10 | 66 |  |

^†^Estimated using chi-square test after excluding missing values

^‡^”Other” category removed due to small numbers

^§^Participant was either unsure whether they had tested or could not place the date with enough precision to unambiguously identify as occurring within the last year
