# Supplementary material for: Harambee!: A pilot mixed methods study of integrated residential HIV testing among African-born individuals in the Seattle area
Source: PLoS One. 2019 May 6;14(5):e0216502. doi: 10.1371/journal.pone.0216502 (PMC6502314; doi:10.1371/journal.pone.0216502)
Supplement: S1 File — (PDF) [file pone.0216502.s002.pdf]

**SUPPORTING INFORMATION**

1) General Questionnaire (self-completed at health care entry)
    a. English
    b. Amharic
    c. Tigrinya
    d. Spanish
    e. Somali
2) HIV Questionnaire (interviewer-completed at point of HIV testing)
3) Key Informant Interview Guide

Study ID label here

**Please answer the following questions. You may choose to leave a question blank if you would prefer not to answer.**

1) How old are you? ____________________

2) What is your gender? ☐ Male ☐ Female ☐ Other:____________

3) Do you identify as transgender? ☐ Yes ☐ No

4) What is your race? **Check all that apply.**
   - ☐ Black/African-American/African
   - ☐ Asian or Asian American
   - ☐ American Indian or Alaska Native
   - ☐ Pacific Islander or Native Hawaiian
   - ☐ Hispanic/Latinx
   - ☐ White
   - ☐ Other: ____________________

5) What is your religious affiliation?
   - ☐ Christian
   - ☐ Muslim
   - ☐ Buddhist
   - ☐ Hindu
   - ☐ Jewish
   - ☐ No religious affiliation
   - ☐ Other: ____________________

6) Are you currently employed? ☐ Yes ☐ No

7) What is the highest level of schooling you have completed?
   - ☐ Primary school
   - ☐ High/secondary school
   - ☐ Some college, but no degree
   - ☐ College degree (e.g. Bachelor's, Master's or professional)
   - ☐ Other: ____________________

8) What language(s) do you primarily speak at home? **Check all that apply.**
   - ☐ English
   - ☐ Spanish
   - ☐ French
   - ☐ Amharic
   - ☐ Somali
   - ☐ Oromiffa
   - ☐ Tigrinya
   - ☐ Arabic
   - ☐ Kiswahili
   - ☐ Other: ____________

9) What zip code do you currently live in? ____________________

10) What country were you born in? ____________________

11) What year did you emigrate to the US? ____________________ ☐ N/A

12) Have you traveled outside of the US in the last 5 years? ☐ Yes ☐ No

13) Do you have any kind of health care coverage, including health insurance, prepaid plans such as HMOs, government plans such as Medicare, or Indian Health Service?

- ☐ Yes
- ☐ No
- ☐ Don't know

14) Do you have one or more persons who you think of as your personal doctor or health care provider?

- ☐ Yes
- ☐ No
- ☐ Don't know

**15) A routine checkup** is a general physical exam, not an exam for a specific injury, illness, or condition. About how long has it been since you last visited a doctor, nurse practitioner, or other health provider **for a routine checkup**?

- ☐ Less than 1 year ago
- ☐ 1-2 years ago
- ☐ 2-5 years ago
- ☐ More than 5 years ago
- ☐ Don't know
- ☐ Never

16) Over the past 2 weeks, **how often** have you had little interest or pleasure in doing things?

- ☐ Not at all
- ☐ Several days
- ☐ More than half of days
- ☐ Nearly every day

17) Over the past 2 weeks, **how often** have you been feeling down, depressed, or hopeless?

- ☐ Not at all
- ☐ Several days
- ☐ More than half of days
- ☐ Nearly every day

18) Have you ever lived in the same house with someone who was sick with tuberculosis (TB)?

- ☐ Yes
- ☐ No
- ☐ I don't know

19) Have you ever taken any medications for tuberculosis (TB) treatment?

- ☐ Yes
- ☐ No
- ☐ I don't know

20) Some people who want to be tested for HIV decide not to get tested because of certain barriers. **Check the one LARGEST barrier that your community faces for HIV testing.**

- ☐ Getting tested is too expensive
- ☐ Don't know where to get tested
- ☐ Testing is too far away
- ☐ Worried about privacy or confidentiality
- ☐ Don't want to know their results
- ☐ Treatment is too expensive
- ☐ Worried about losing their partner
- ☐ Worried about losing their job
- ☐ No barriers
- ☐ Other: ________________________

QUESTIONNAIRE PART I (AMHARIC) Study ID label here

**የሚከተሉትን ጥያቄዎች እባኮትን ይመልሱ። ጥያቄውን መመለስ ካልፈለጉ መልሱን ባዶ መተው ትችላሉ።**

1) እድሜዎ ስንት ነው? ______________________

2) ፆታዎ ምንድን ነው? ☐ ወንድ ☐ ሴት ☐ ሌላ፥

3) ራሶን እንደ ትራንስጀንደር (ፆታ የለወጠ) ይቆጥራሉ? ☐ አዎ ☐ አይ

4) የእርስዎ ዘር ምንድን ነው? **የሚመለከቶትን ሁሉ ይምረጡ።**

☐ ጥቁር/አፍሪካ አሜሪካዊ/አፍሪካዊ
☐ እስያዊ ወይም እስያ አሜሪካዊ
☐ ሕንድ አሜሪካዊ ወይም የአላስካ ተወላጅ
☐ የፓስፊክ ደሴት ነዋሪ ወይም የሃዋይ ተወላጅ
☐ የእስፓንኛ/ላቲን
☐ ነጭ
☐ ሌላ፥ ______________________

5) ሃይማኖትዎ ምንድን ነው?

☐ ክርስቲያን
☐ ሙስሊም
☐ ቡድሂስት
☐ የሂንዱ እምነት
☐ አይሁዳዊነት
☐ የሃይማኖት አባልነት የለኝም
☐ ሌላ ______________________

6) በአሁን ሰዓት ስራ ይሰራሉ? ☐ አዎ ☐ አይ

7) የመጨረሻ ከፍተኛ የትምህርት ደረጃ የጨረሱት?

☐ የመጀመሪያ ደረጃ ትምህርት ቤት
☐ ሁለተኛ ደረጃ ትምህርት ቤት
☐ ኮሌጅ ያለ ዲግሪ
☐ ኮሌጅ በዲግሪ (ምሳሌ፥ የመጀመሪያ ፣ ሁለተኛ ፣ ወይም የሞያ ዲግሪ)
☐ ሌላ፥______________________

8) የትኛውን ቋንቋ ነው ቤት ውስጥ የሚናገሩት? **የሚመለከትዎት ሁሉ ላይ ክብ ያድርጉ።**

☐ እንግሊዝኛ
☐ ስፓንኛ
☐ ፈረንሳይኛ
☐ አማርኛ
☐ ሶማሊኛ
☐ ኦሮምኛ
☐ ትግሪኛ
☐ አረብኛ
☐ ኪስዋሊኛ
☐ ሌላ፥ ______________

9) የአካባቢያዊ መለያ ቁጥሮ (ወይም zip code) በአሁን ጊዜ ምንድን ነው? ______________________

10) የት አገር ነው የተወለዱት? ______________________

11) በስንት ዓመተ ምህረት ነው ወደ አሜሪካ የመጡት? ______________________ ☐ N/A

12) ባለፈው አምስት አመት ወስጥ ከአሜሪካ ውጪ ጉዞ ሄደዋል? ☐ አዎ ☐ አይ

13) የጤና ኢንሹራንስ እንደ HMO ቅድሞ ክፍያ ፕሮግራም ፣ ወይም  የመንግስት ፕሮግራም እንደ Medicare ፣ ወይም የሕንድ ጤና አገልግሎት (Indian Health Service) ፣ ወይም ሌላ የጤና ጥበቃ ሽፋን አላችሁ ?

☐ አዎ ☐ አይ ☐ አላውቅም

14) የግል ሐኪም ወይም የጤና አገልግሎት ሰጪ የምትሉት ቢያንስ አንድ ሰው አላችሁ?

☐ አዎ ☐ አይ ☐ አላውቅም

15) **መደበኛ ምርመራ** ማለት የተለየ ጉዳት ፣ ህመም ፤ ወይም ሁኔታ ሳይሆን በአጠቃላይ አካላዊ ምርመራ ነው። ስለዚህ ምን ያህል ጊዜ ሆንዎታል እርሶ በሃኪም ፣ ነርስ ፣  ወየም ሌላ ጤና  አገልግሎት ሰጪ **መደበኛ ምርመራ** የተደረጉት?

☐ ከአንድ አመት በታች
☐ ከሁለት እስከ አምስት አመት በፊት
☐ አላውቅም
☐ ከአንድ እስከ ሁለት አመት መሃል
☐ ከአምስት አመት በላይ
☐ በጭራሽ መቼም

| | |
|---|---|
| 16) በአለፈው ሁለት ሳምንት ውስጥ ስራዎችን ለመስራት ፍላጎት ማጣት ወይም  ደስታ ማጣት ያጋጠሞት ስንት ጊዜ ይሆናል? | ☐ በጭራሽ መቼም<br>☐ ብዙ ቀናት<br>☐ ግማሽ ቀናት በላይ<br>☐ በየቀኑ ማለት ይቻላል |
| 17) በአለፈው ሁለት ሳምንት ውስጥ ፣ ስንት ጊዜ ይሆናል ተስፋ መቁረጥ ወይም ተስፋ የመቁረጥ ስሜት ያጋጠመዎት? | ☐ በጭራሽ መቼም<br>☐ ብዙ ቀናት<br>☐ ግማሽ ቀናት በላይ<br>☐ በየቀኑ ማለት ይቻላል |

18) ቲዩበርክሎዝስ (tb) በሽታ ከያዘው ሰው ጋር አብራችሁ በአንድ ቤት ውስጥ ኖራችሁ ታውቃላችሁ?

☐ አዎ ☐ አይ ☐ አላውቅም

19) ለቲዩበርክሎዝስ (tb) ሕክምና መድሃኒት ወስዳችሁ ታውቃላችሁ?

☐ አዎ ☐ አይ ☐ አላውቅም

20) አንዳንድ ሰዎች ኤች አይ ቪ (HIV) ምርመራ እየፈለጉ በተለያየ ምክንያት ላለመፈተን ይወስናሉ። **በእርሶ ህብረተሰብ ለ**HIV **ላለመመርመር ትልቁ ምክንያት ወይም መሰናክል ይህ ነው የሚሉትን ክብ ያድርጉ።**

☐ ምርመራው ውድ ነው
☐ የት እንደሚመረምሩ አለማወቅ
☐ የምርመራው ቦታ ሩቅ ስለሆነ
☐ ስለ ግላዊነት ወይም ሚስጥርነት በመስጋት
☐ የምርመራውን ውጤት ማወቅ ስለማይፈለግ
☐ ሕክምናው ውድ ስለሚሆን
☐ የሕይወት አጋርን ላለማጣን በመስጋት
☐ ስራን ላለማጣት በመስጋት
☐ መሰናክል የለም
☐ ሌላ፥ ____________________

**በይዘኦም እዞም ጥያቄታት ይመልሱ. ምምላስ ተዘይደኣ ደሊዮም ነጻ ምግዳፍ ይኽእሉ እዮም።**

1) ክንደይ ዓመትኻ/ኺ? ______________________

2) ጾታ? ☐ ተባዕታይ ☐ ኣንስታይ ☐ ካልእ: ______________________

3) ትራንስጀንደር ኢኻ/ኺ ክትባሃል ይካኣል'ዶ? (ጾታ ዝለወጥካ) ☐ እወ ☐ ኣይኾንኩን

4) ዘርእኻ/ኺ እንታይ እዩ? **መልሲ ትብሎ ኩሉ ኣኽብብ.**
- ☐ ጸሊም/ኣፍሪካንኣሜሪካን/ኣፍሪካዊ
- ☐ ኤሲያዊ ወይ ኤስያን ኣሜርካን
- ☐ ኤሲያዊ ወይ ኤስያን ኣሜርካን
- ☐ ፐስፊክ ኢስላንደር ወይ ወዲባት ሁዋያን
- ☐ ሂስፖኒክ/ላቲናዊ
- ☐ ጻዕዳ
- ☐ ካልእ: ______________________

5) ሃይማኖትካ/ኪ እንታይ እዩ?
- ☐ ክርስትያን
- ☐ ሙስሊም
- ☐ ቡዲስት
- ☐ ሂንዱ
- ☐ ጂዊሽ
- ☐ ምንንም ሃይማኖት ዘይከተል
- ☐ ካሊኣ: ______________________

6) ኸዚ ዝተቐጸርካዮ ስራሕ ኣለካ'ዶ? ☐ እወ ☐ የብለይን

7) ዝላዓለ ትምህርቲ ደረጃካ/ኪ ክነደይ እዩ?
- ☐ ፕራይሜሪ ስኩል
- ☐ ሃይ/ሰከንደሪ ስኩል
- ☐ ኮሌጅ፣ ብዘየ ዲግሪ
- ☐ ኮሌጅ ዲግሪ (ናኣብነት፡- ናይ መጀመርታ ዲግሪ፣ ማስተርስ ፣ ፕሮፌሺናል)
- ☐ ካልእ: ______________________

8) ኣብ ገዛ ኣብሊጽኻ/ኺ እትጥቀመሉ ቋንቋ/ቋንቋታት? **መልሲ ትብሎ ኣኽብብ.**
- ☐ ኢንግሊሽ
- ☐ ስፓኒሽ
- ☐ ፍሬንች
- ☐ ኣማሪኛ
- ☐ ሶማል
- ☐ ኦሮሚፋ
- ☐ ትግርኛ
- ☐ ዓረብ
- ☐ ኪስዋሂሊ
- ☐ ካልእ: ______________________

9) ኸዚ ኣበየናይ ዚፕ ኮድ ትነብር?______________________

10) ኣበይናይ ሃገር ተዎሊድኻ/ኺ? ______________________

11) ናብ ዩ ኤስ ኤ መዓዝ ኣቲኻ/ኺ? ______________________ ☐ N/A

12) ኣብዚ 5 ዓመት እዚ ካብ ዩ ኤስ ኤ ወጺኻ ኔርካ ዶ? ☐ እወ ☐ ኣይወጻኩን

13) ዝኾነ ዓይነት ናይ ሕክምና ግልጋሎት ሽፋን ኣለካ ዶ? ከም ናይ ሕክምና ኢንሹራንስ ፣ ከም ቅድመ ክፍሊት ፕላን HMOs፣ መንግስታዊ ፕላን ከም ሜዲኬር ወይ ኢንድያን ሕክምና ግልጋሎት

☐ እወ ☐ የብለይን ☐ ኣይፈልጥን

14) ሓደ ወይ ካብኡ ንላዕሊ ግላዊ ዶክተር ወይ ናይ ሕክምና ግልጋሎት ዝህብ ኢልኻ ትሓስቦ ሰብ ኣለካ'ዶ?

☐ እወ ☐ የብለይን ☐ ኣይፈልጥን

15) **መደበኛ ምርመራ** ጠቕላላ ናይ ኣካላዊ ምርመራ እዩ፤ ን ኣብነት ከም ንጹር ጉዳኣት፣ ሕማመ ወይ ጊዛዊ ሁውከት ኣይኮነን። ዶክተር፣ ነርስ ወይ ናይ ሕክምና ግልጋሎት **መደበኛ ምርመራ** ካብዘይትረክብ ኽንደይ ጌርኻ?

☐ ቅድሚ ትሕቲ 1 ዓመት ☐ ቅድሚ 2-5 ዓመት ☐ ኣይፈልጥን
☐ ቅድሚ 1-2 ዓመት ☐ ቅድሚ 5 ዓመት ብላዓሊ ☐ በፍጹም

16) ካብቲ ዝሓለፈ 2 ቅነ ብላዕሊ ክንደየናይ ግዜ እዩ ንእሽተይ ድሌት ወይ ንእሽተይ ነገራት ናይ ምግባር ሓጐስ ዝነበረካ?

☐ በፍጹም
☐ ዝተወሰኑ መዓልቲታት
☐ ካብ ፍርቂ መዓልቲ ብላዓሊ
☐ ዳርጋ ኩሉ መዓልቲ

17) ካብቲ ዝሓለፈ 2 ቅነ ብላዕሊ ክንደየናይ ግዜ እዩ ናይ ትሕተኻንት ስሚዒት, ድብርት, ወይ ተስፋቖራጽነት ችግር ዝርብሽካ ዝነበረ?

☐ በፍጹም
☐ ዝተወሰኑ መዓልቲታት
☐ ካብ ፍርቂ መዓልቲ ብላዓሊ
☐ ዳርጋ ኩሉ መዓልቲ

18) ብ ቲቦርክሎሲስ(ቲቢ) ሓሚሙ ዝነበረ ኣካል?

☐ እወ ☐ ኣይኮነን ☐ ኣይፈልጥን

19) ንቲቦርክሎሲስ(ቲቢ) ዝኸውን ዝኾነ ዓይነት ፈውሲወሲዶም ይፈልጡ ዶ?

☐ እወ ☐ ኣይኮነን ☐ ኣይፈልጥን

20) ዝተወሰኑ ኤች ኣይ ቪ ክምርመሩ ዝደልዩ ሰባት ብዝተፈላለዩ መሰናኽል ንከየምርመሩ ወሲኖም፡፡. **ኤች ኣይ ቪ ንኽይትምርመር ንህዝብኻ እቲ ዝዓበየ መሰናክል ዝኾነ ኣኽብብ .**

☐ ምምርማር ብጥዕሚ ክባር ስለዝኮነ
☐ ናይ ምርመራ ቦታ ዘይምፍላጥ
☐ ናይ ምርመራ ቦታ ብምርሓቑ
☐ ሚስጥራዉነተይ ንምሕላው
☐ ዉጺእተይ ክፈልጥ ስለ ዘይድሊ
☐ ምሕካሙ ብጥዕሚ ክባር ስለዝኮነ
☐ ፍቕረኛኦም ከይስእኑ ብምፍራሕ
☐ ስራሖም ከይስእኑ ብምፍራሕ
☐ መሰናክል የለን
☐ ካልእ: ______________________

**Por favor responda a las siguientes preguntas. Puede dejar la respuesta vacía, si prefiere no responder.**

1) ¿Qué edad tiene usted? ______________________________

2) ¿Cuál es su género? ☐ Hombre ☐ Mujer ☐ Otro:____________________

3) ¿Se identifica usted como transgénero? ☐ Sí ☐ No

4) ¿Cuál es su raza? **Marque con un círculo todas las opciones aplicables.**

- ☐ Negro/afroestadounidense/africano
- ☐ Asiático o asiático estadounidense
- ☐ Indígena estadounidense o nativo de Alaska
- ☐ Isleño del Pacífico o hawaiano nativo
- ☐ Hispano o latino
- ☐ Blanco
- ☐ Otro: ______________________________

5) ¿Cuál es su afiliación religiosa?

- ☐ Cristiano
- ☐ Musulmán
- ☐ Budista
- ☐ Hinduista
- ☐ Judío
- ☐ Ninguna afiliación religiosa
- ☐ Otro: ______________________________

6) ¿Tiene usted un empleo actualmente? ☐ Sí ☐ No

7) ¿Cuál es el nivel educativo más alto que usted ha completado?

- ☐ Escuela primaria (entre 1 a 6 años)
- ☐ Escuela secundaria (entre 7 a 12 años)
- ☐ Algunos años de universidad pero no obtuvo el título
- ☐ Título de pregrado o de postgrado (ejemplos: títulos de cuatro años, de maestría o profesionales)
- ☐ Otro: ______________________________

8) ¿Qué idioma(s) habla principalmente en su casa? **Marque con un círculo todas las opciones aplicables.**

- ☐ Inglés
- ☐ Español
- ☐ Francés
- ☐ Amhárico
- ☐ Somalí
- ☐ Oromo
- ☐ Tigrinya
- ☐ Árabe
- ☐ Kiswahili
- ☐ Otro: ____________

9) ¿En qué código postal vive usted actualmente? ______________________________

10) ¿En qué país nació usted? ______________________________

11) ¿En qué año emigró usted a los Estados Unidos? ______________________________ ☐ N/A

12) ¿Ha viajado fuera de los Estados Unidos en los cinco últimos años? ☐ Sí ☐ No

13) ¿Tiene usted algún tipo de cobertura de atención de la salud, incluyendo un seguro médico, planes prepagados tales como HMO, planes de gobierno tales como Medicare o el Servicio de Salud Indígena?

- ☐ Sí
- ☐ No
- ☐ No sé

14) ¿Tiene usted a una o más personas que considere como médico personal o proveedor de atención de la salud?

- ☐ Sí
- ☐ No
- ☐ No sé

15) **Un chequeo rutinario de salud** es un examen físico general y no un examen de una lesión, enfermedad, ni condición específica. ¿Hace cuánto tiempo aproximadamente visitó usted por última vez a un médico, enfermera especializada u otro proveedor de salud **para un chequeo rutinario de salud**?

- ☐ Hace menos de un año
- ☐ Hace uno a dos años
- ☐ Hace dos a cinco años
- ☐ Hace más de cinco años
- ☐ No sé
- ☐ Nunca

16) ¿En las últimas dos semanas, con cuánta frecuencia ha tenido usted poco interés o disfrute al hacer cosas?

- ☐ Para nada
- ☐ Varios días
- ☐ Más de la mitad de los días
- ☐ Casi todos los días

17) ¿En las últimas dos semanas, con cuánta frecuencia se ha sentido usted decaído, deprimido o sin esperanza?

- ☐ Para nada
- ☐ Varios días
- ☐ Más de la mitad de los días
- ☐ Casi todos los días

18) ¿Ha vivido alguna vez en la misma casa con alguna persona enferma de tuberculosis (TB)?

- ☐ Sí
- ☐ No
- ☐ No sé

19) ¿Ha tomado alguna vez algún medicamento para tratar la tuberculosis (TB)?

- ☐ Sí
- ☐ No
- ☐ No sé

20) Algunas personas que desean realizarse la prueba de VIH deciden no hacerlo debido a ciertas barreras. **Marque un círculo alrededor de la PRINCIPAL barrera que su se enfrenta su comunidad en cuanto a pruebas de VIH.**

- ☐ Las pruebas son demasiado caras.
- ☐ No saben dónde realizarse las pruebas.
- ☐ Los lugares para las pruebas son muy lejos.
- ☐ Preocupación sobre la privacidad o la confidencialidad.
- ☐ No quieren saber los resultados.
- ☐ El tratamiento es demasiado caro.
- ☐ Preocupación de perder a la pareja.
- ☐ Preocupación de perder el trabajo.
- ☐ No hay barreras
- ☐ Otro: ____________________

**Fadlan ka jawaab su'aalahan soo socda. Waxa ka tagi kartaa su'aal adigoon ka jawaabin haddii aad doontid in aadan ka jawaabin.**

1) Imisa jir ayaad tahay? ______________________

2) Muxuu yahay jinsigaagu? ☐ Lab ☐ Dheddig ☐ Mid Kale:______________

3) Miyaad isu aqoonsantahay jinsigaaga inaad ka duwan tahay? ☐ Haa ☐ Maya

4) Maxay tahay qowmiyaddaadu? **Goobaab dhammaan wixii khuseeya.**

- ☐ Madow/Afrikan-Amerikaan/Afrikaan
- ☐ Aasiyaan ama Ameerikaan Aasiyaan
- ☐ Hindi Ameerikaan ama Dhaladka Alaska
- ☐ Pacific Islander ama Dhaladka Hawaiian
- ☐ Hispanic/Latino
- ☐ Caddaan
- ☐ Mid Kale: ______________________

5) Maxay tahay caqiidadaada diineed??

- ☐ Kiristaan
- ☐ Muslim
- ☐ Buddhist
- ☐ Hindu
- ☐ Yuhuud
- ☐ Ma jirto Caqiido diineed
- ☐ Mid Kale: ______________________

6) Miyaad hadda shaqaysaa? ☐ Haa ☐ Maya

7) Waa maxay heerka ugu sarreeya dugsiga ee aad dhammaysay?

- ☐ Dugsi hoose
- ☐ Dugsi sare
- ☐ Xoogaa kulliyad ah, laakiin bilaa shahaado jaamacadeed
- ☐ Shahaado Jaamacadeed (sida, Shahaadada Koowaad, Shahaado Bajulaarka, Master ah ama shahaado Mihnadle ah)
- ☐ Mid Kale: ______________________

8) Maxay tahay luqadda koowaad ee aad kaga hashasho guriga? **Goobaab dhammaan wixii khuseeya.**

- ☐ Ingiriisi
- ☐ Isbaanish
- ☐ Faransiis
- ☐ Amharic
- ☐ Somali
- ☐ Oromiffa
- ☐ Tigrinya
- ☐ Carabi
- ☐ Kiswahili
- ☐ Mid Kale: __________

9) Muxuu yahay zip code-ka aad hadda ku nooshahay?

10) Wadankee ayaad ku dhalatay? ______________________

11) Sannadkee ayaad u soo guurtay Maraykanka? ______________________ ☐ N/A

12) Ma u safartay meel ka baxsan Maraykanka 5tii sanno ee la soo dhaafay? ☐ Haa ☐ Maya

13) Miyaad leedahay nooc caymis daryeel caafimaad, oo ay ku jiraan caymiska caafimaadka, qorshayaashii horay loo bixiyay sida HMOs, qorshayaasha dawladda sida Medicare, ama adeegga "Indian Health Service"?

☐ Haa ☐ Maya ☐ Ma aqaan

14) Miyaad leedahay qof ama ka badan oo aad u malaynayso inuu yahay dhakhtarkaaga gaarka ah ama adeeg-bixiyahaaga xanaanada caafimaadka?

☐ Haa ☐ Maya ☐ Ma aqaan

15) **Baaritaan caafimaad oo joogta ah** waa baaritaan guud oo jireed, ma'aha baaritaan u gaar ah dhaawac, jirro, ama xaalad. Muddo intee dhan ah ayaa laga joogaa markii ugu dambeysay ee aad soo booqatay dhakhtar, kalkaaliye sare oo caafimaad, ama bixiye caafimaad oo kale, **si laguugu sameeyo baaritaan caafimaad oo joogta ah**?

- ☐ Wax ka yar 1 sanno kahor
- ☐ 1-2 sanno kahor
- ☐ 2-5 sanno kahor
- ☐ In kabadan 5 sanno kahor
- ☐ Ma aqaan
- ☐ Ma jiro

16) 2-dii toddobaad ee ugu dambeeyay, meeqa jeer ayaad dareentay daneyn yar ama raaxo-darro inaan wax qabto?

- ☐ Marnaba
- ☐ Maalmo dhowr ah
- ☐ In ka badan nus maalin
- ☐ Ku dhawaad maalin kasta

17) 2-dii toddobaad ee ugu dambeeyay, meeqa jeer ayaad dareentay hoose u dhac, niyad jab, ama rajo la'aan?

- ☐ Marnaba
- ☐ Maalmo dhowr ah
- ☐ In ka badan nus maalin
- ☐ Ku dhawaad maalin kasta

18) Weligaa guri ma kula nooleed qof qaba cudurka qaaxada ama "TB"da?

☐ Haa ☐ Maya ☐ Ma aqaan

19) Weligaa ma qaadatay daawada cudurka qaaxada ama "TB"da?

☐ Haa ☐ Maya ☐ Ma aqaan

20) Dadka qaarkood oo raba in laga baaro HIV-ga waxay go'aansadaan inaan la baarin sababta oo ah caqabado gaar ah. **Goobaab caqabadda UGU WEYN ee komuyuunatigaagu wajahdoin laga baaro HIV.**

- ☐ Sameynta baaritaanku waa qaali
- ☐ Ma yaaqaanaan meesha laga qaato baaritaanka
- ☐ Baaritaanku waa mid aad u fog
- ☐ Walwel ku saabsan qarsoodiga iyo gaar-ahaanta macluumaadkeyga
- ☐ Ma rabaan ina y ogaadaan natiijadooda
- ☐ Dawaynta aad ayay qaali u tahay
- ☐ Walwal la xiriira wajahaadda lammaanahooda
- ☐ Walwal la xirira waayista shaqadooda
- ☐ Ma jiraan caqabado
- ☐ Mid Kale:
- ☐ ________________________

Start Time: ________________ End Time: ________________

**QUESTIONNAIRE PART II**

Study ID label here

**INTERVIEW LANGUAGE (circle one)**

English Amharic Tigrinya Somali Other:________________________

***Begin by examining the participant checklist. If the health fair participant is participating in the study, the participant must have the informed consent stamped to undergo this questionnaire. Before asking questions, inform the participant that this is a short, anonymous questionnaire about HIV testing. They can decline to answer any question that makes them uncomfortable.***

| **HIV Section: To be completed in person by HIV tester**<br>**Ask these questions to all individuals before offering an HIV test** | |
|---|---|
| Have you ever been tested for HIV? | ☐ Yes<br>☐ No |
| **If you have tested before**, when was the last time you were tested? | Year: ______________________<br>Month (if known): ______________________ |
| **If you have tested before,** in what country did you receive your last HIV test? | ☐ USA<br>☐ Outside of the USA (write in country)<br>________________________________ |
| **If you have tested before**, where did you receive your last HIV test? | ☐ Hospital, clinic, or other health facility<br>☐ Community organization (e.g., CMCH)<br>☐ Health fair (e.g., Key Arena) or other outreach event<br>☐ Immigrant health exam<br>☐ Other<br>______________________________________ |
| **If you have tested before**, what was the result of your last HIV test? | ☐ Negative<br>☐ Positive<br>☐ Don’t know<br>☐ Decline to answer |
| **If you have tested POSITIVE before,** are you currently on treatment? | ☐ No<br>☐ Yes |
| **Refer client to One-on-One if not on treatment. Comment on treatment status in the box at the end of the questionnaire.** | |
| How many sexual partners have you had in the last 12 months? | |
| | |
| Do you have a friend or family member living with HIV? | ☐ Yes<br>☐ No |

| | |
|---|---|
| What barriers, if any, do you face in getting tested for HIV? [**Do not read the list of barriers** to client**– instead, check all reasons spontaneously provided by the client.]** | ☐ I don't have transportation to the site<br>☐ I don't know where to go for testing<br>☐ I don't have enough time<br>☐ The testing site is too far away<br>☐ I don't like the people at the testing sites<br>☐ I am worried about confidentiality<br>☐ I might be recognized at the testing site<br>☐ There is no cure so why get tested<br>☐ I can't afford treatment so why get tested<br>☐ I don't want to know my results<br>☐ I am afraid of losing my health insurance<br>☐ I am afraid of losing my job<br>☐ I am afraid of losing my partner or family<br>☐ No barriers<br>☐ Other: ___________________________________ |
| **If the client is not already HIV-positive, ask the client if they would like to test for HIV today. Follow standard U-TEST protocols.** | |
| Did the client choose to test? | ☐ Yes<br>☐ No |
| If no test, why did the client decide not to test? | ☐ Already knows HIV status<br>☐ Concerned about confidentiality<br>☐ Does not feel at risk for HIV infection<br>☐ Other: _________________________________ |
| **For in-person testing only:** What was the test result? | ☐ Reactive<br>☐ Non-reactive<br>☐ Invalid (client did not receive a test result today)<br><br>Lot Number:<br><br>Expiration Date: |
| **General Comments or Observations** | |

***Once you have completed documenting the participant responses above, please stamp the "Questionnaire II" part of their checklist. Do not check whether or not they have tested for HIV.***

**Key Informant Interview Guide** Today's date: _________ Interviewer: ________________

## A. Background information

Age: ______________________________ Gender (circle one): Female Male Other

Country of birth: ____________________ Primary language: ________________________________

Occupation: ________________________ Health board/organization affiliation: _________________

## B. General healthcare

| Question/topic area | Responses/notes |
|---|---|
| 1) What are the biggest barriers for African immigrants in engaging in preventative healthcare in the Seattle area?<br>- How improve?<br>- Are there any ways around these barriers that people in your community/culture have used? How successful was this? | |
| 2) What aspects of the healthcare system could be changed or improved to help African immigrants be better engaged in preventative healthcare?<br>- What have you heard or noticed how members of your community/culture are generally treated in formal healthcare settings (clinics, hospital, etc.?) | |
| 3) In your community/culture, which member of the family member tends to coordinate healthcare needs for the family?<br>- What works well about this structure? What is difficult?<br>- Are children or adults more likely to see the doctor and/or dentist more regularly? | |
| 4) Do you feel that men or women are more likely to seek preventative healthcare in your community/culture?<br>- What works well about this structure? What is difficult?<br>- How do women find care during pregnancy and childbirth? What have you experienced or heard about women's experiences with healthcare providers during pregnancy and childbirth? How do you feel this experience would be different if a woman was known to be HIV positive? | |
| 5) Are there any alternative healthcare providers that are popular in your community/culture?<br>- Who has access to these providers, and how?<br>- How do people go about paying these providers?<br>- What kinds of services do they provide?<br>- Are there many practicing or non-practicing mainstream healthcare providers in your community who provide care for other community members? | |
| 6) What types of locations are convenient for your community members to access healthcare services? How likely are people in your community/culture to use healthcare services offered in or near their apartment building? | |

**C. Health fair/HIV**

| Question/topic area | Responses/notes |
|---|---|
| 1) What did you like about the Harambee! mini-health fair?<br>- List three things that worked well? | |
| 2) How do you think the mini-health fairs can be improved?<br>- List three things that could be improved upon? | |
| 3) Are there any unique aspects of your community/culture that worsen or improve stigma associated with seeking HIV testing or care?<br>- How do you think members of your community/culture would feel about a family member or friend testing positive for HIV?<br>- How do you think an HIV positive person from your community/culture would be perceived or treated if they shared their diagnosis with another member of their community? with their regular healthcare provider or a new healthcare provider? Is there anything in particular they would not be able to participate in? | |
| 4) In your opinion, what are the biggest barriers to African immigrants in the Seattle area getting tested for HIV?<br>- What do you think would help to improve those things?<br>- Are there any ways around these barriers that people in your community/culture have used? How successful was this? | |
| 5) What would your ideal setting for HIV testing look like?<br>- What fears do you think community members would have about getting HIV testing in a community center? In a regular clinic or doctor's office? At home with a test kit?<br>- Do you foresee any issues with providing HIV testing and other healthcare services where many members of your community/culture live? | |
| 6) Did you get tested for HIV at or after the health fair? Why or why not?<br>- If you did get tested, did you use the provider-offered test or the self-test kit? How was your experience using that modality? Would you recommend that modality to someone else? | |
| 7) Do you consider yourself at risk for getting HIV? Do you consider others you know at risk for getting HIV? If so, how or why?<br>- Do you travel outside of the US or back to your home country? If so, where do you go? Do you ever engage in sex there? How many sexual partners might you have on a typical trip there? | |
| Other comments or observations: | |
